# Clinicopathological Characteristics and Treatment Patterns of Extremity, Trunk, and Axial Sarcomas: A Descriptive Real-World Cohort Study from a Romanian Tertiary Oncology Center

**DOI:** 10.3390/diagnostics16111659

**Published:** 2026-05-28

**Authors:** Mihai-Teodor Georgescu, Adelina Silvana Gheorghe, Romina-Marina Sima, Bashar Haj Hamoud, Georgia Luiza Serbanescu, Andreea Veronica Lazescu, Oana Gabriela Trifanescu, Radu Iulian Mitrica, Laurentia Nicoleta Gales, Roxana Rahnea-Nita, Andra-Elena Balcangiu-Stroescu

**Affiliations:** 1 Oncology 2 Discipline, Department 8—Radiology, Oncology, Hematology, Faculty of Medicine, “Carol Davila” University of Medicine and Pharmacy, Bulevardul Eroii Sanitari 8, 050474 Bucharest, Romania; mihai.georgescu@umfcd.ro (M.-T.G.); luiza.serbanescu@umfcd.ro (G.L.S.); andreea.lazescu@umfcd.ro (A.V.L.); oana.trifanescu@umfcd.ro (O.G.T.); radu.mitrica@umfcd.ro (R.I.M.); laurentia.gales@umfcd.ro (L.N.G.); 2 Department of Medical Oncology I, “Prof. Dr. Alexandru Trestioreanu”, Institute of Oncology, 022328 Bucharest, Romania; adelina.silvana.gheorghe@gmail.com; 3 Department of Obstetrics and Gynecology, “Carol Davila” University of Medicine and Pharmacy, 020021 Bucharest, Romania; 4 Department for Gynecology, Obstetrics and Reproductive Medicine, Saarland University Hospital, 66421 Homburg, Germany; bashar.hajhamoud@uks.eu; 5 Oncology 1 Discipline, Department 8—Radiology, Oncology, Hematology, Faculty of Medicine, “Carol Davila” University of Medicine and Pharmacy, Bulevardul Eroii Sanitari 8, 050474 Bucharest, Romania; roxana.rahnea-nita@umfcd.ro; 6 Discipline of Physiology, Department 3, Faculty of Stomatology, “Carol Davila” University of Medicine and Pharmacy, 020021 Bucharest, Romania; andra.balcangiu@umfcd.ro

**Keywords:** soft tissue sarcoma, bone sarcoma, extremity sarcoma, overall survival, Kaplan–Meier, multimodal treatment, osteosarcoma, undifferentiated pleomorphic sarcoma, Romania, Eastern Europe

## Abstract

**Background and Objectives**: Sarcomas are rare malignancies of mesenchymal origin comprising less than 1% of all adult solid tumors, exhibiting marked histological heterogeneity and variable clinical behavior. Data from Eastern European tertiary oncology centers remain scarce. This study characterized the clinicopathological features, treatment modalities, and survival outcomes of patients with sarcomas of the extremities and trunk treated at the “Prof. Dr. Alexandru Trestioreanu” Institute of Oncology from Bucharest over a ten-year period. **Materials and Methods**: We conducted a retrospective analysis of 164 patients diagnosed with sarcomas of the extremities and trunk between 2010 and 2020 at “Prof. Dr. Alexandru Trestioreanu” Institute of Oncology. Variables included age, sex, tumor localization, histological subtype, immunohistochemical profile, treatment modalities, recurrence, metastatic spread, and overall survival (OS). Kaplan–Meier curves estimated survival; log-rank tests were applied for subgroup comparisons. **Results**: The cohort comprised 82 males and 82 females (50.0% each), with a mean age of 48.8 ± 18.3 years. The lower limb was the most frequent site (*n* = 96, 58.5%), particularly the thigh/femur (34.1%). The most common subtypes were undifferentiated pleomorphic sarcoma (14.6%), osteosarcoma (12.2%), and fibrosarcoma (11.0%). Surgery was performed in 75.6%, chemotherapy in 80.5%, and radiotherapy in 59.8%. Local recurrence occurred in 35.4% and distant metastases in 41.5%. The median OS was 96.0 months (vital status known for 160/164 patients; 90 deceased, 70 alive; OS duration available in 126 patients). Metastatic disease was associated with shorter observed survival in descriptive Kaplan–Meier analysis (log-rank *p* < 0.001); this comparison is exploratory given the time-dependent nature of the variable. Survival ranged from 11.5 months (leiomyosarcoma) to 162.5 months (dermatofibrosarcoma protuberans) by histotype. **Conclusions**: This study provides clinically relevant epidemiological and survival data from Romania. The findings illustrate real-world heterogeneity of sarcoma presentations and outcomes at an Eastern European tertiary center and highlight the need for improved diagnostic standardization, prospective data collection, and integration within specialized sarcoma networks.

## 1. Introduction

Sarcomas represent a heterogeneous group of rare malignant tumors of mesenchymal origin, accounting for approximately 1% of all adult solid malignancies and 15% of pediatric cancers [1]. In 2020, the Global Cancer Observatory estimated over 130,000 new cases of bone and soft tissue sarcomas worldwide, with a disproportionately high disease burden in terms of years of life lost [2]. In Europe, the incidence of soft tissue sarcomas (STS) ranges from 4 to 6 per 100,000 individuals per year, while primary bone sarcomas are reported at approximately 0.8–1.0 per 100,000 per year [3].

These tumors may arise from any anatomical site; however, extremities—particularly the lower limbs—account for up to 60% of all STS cases [4]. The WHO classification recognizes more than 100 distinct histological subtypes, each exhibiting unique biological behavior, prognostic implications, and differential sensitivity to therapeutic interventions [5]. This extraordinary heterogeneity renders sarcomas among the most diagnostically and therapeutically challenging tumor families in oncological practice.

The cornerstone of curative treatment for localized extremity sarcomas remains surgical resection with adequate margins [6]. However, local recurrence rates of 20–40% even after margin-negative surgery, and the propensity for distant metastasis—predominantly to the lung—continue to drive significant mortality [7,8]. Chemotherapy and radiotherapy provide complementary therapeutic value depending on histotype, grade, size, and margin status [9,10]. Despite advances in molecular pathology, the five-year OS for high-grade metastatic STS remains below 20% [11,12,13,14,15].

Epidemiological and outcomes data from Eastern European oncology institutions remain substantially underrepresented in the international literature. “Prof. Dr. Alexandru Trestioreanu” Institute of Oncology, established in 1949, is the oldest and largest oncological institution in Romania and constitutes the primary national reference center for sarcoma management. Against this background, the present study was designed as a descriptive retrospective cohort study with the objective of characterizing the patterns of presentation, treatment modalities, and survival outcomes of patients with sarcomas of the extremities, trunk, and axial skeleton treated at this institution over a ten-year period (2010–2020). By documenting real-world institutional experience in an underrepresented regional setting, this study seeks to contribute to the Eastern European sarcoma literature and to provide a benchmark for future prospective data collection.

## 2. Materials and Methods

### 2.1. Study Design and Patient Selection

This retrospective cohort study included all patients diagnosed with histologically confirmed sarcomas of the extremities or trunk treated at “Prof. Dr. Alexandru Trestioreanu” Institute of Oncology between January 2010 and December 2020. Inclusion criteria were: (1) histologically confirmed primary bone or soft tissue sarcoma; (2) primary tumor localized to the extremities, trunk, or axial skeleton; (3) age ≥ 1 year at diagnosis; and (4) availability of clinical follow-up data for at least one post-treatment evaluation.

### 2.2. Data Collection

Clinicopathological data were systematically extracted from institutional medical records. Variables included: sex; age at diagnosis; anatomical site; histological diagnosis; tumor size; immunohistochemical panel results; comorbidities; treatment modalities (surgical procedure, chemotherapy regimen, radiotherapy doses and fractionation); timing and site of local recurrence; metastatic sites; second primary malignancies; and vital status at last follow-up. Overall survival (OS) was defined as the interval in months from the date of histological diagnosis to death from any cause, or to the date of last documented contact for surviving patients (censored observations). OS data were available for 126 of 164 patients (76.8%); the remaining 38 patients lacked sufficient follow-up documentation.

### 2.3. Histological Classification

All diagnoses were established on histopathological examination of biopsy or resection specimens, supplemented by immunohistochemistry in 72.0% of cases (*n* = 118). Classification followed the fifth edition of the WHO Classification of Tumors of Soft Tissue and Bone (2020) [5]. Tumors were further grouped into bone sarcomas (osteosarcoma, Ewing sarcoma, chondrosarcoma; *n* = 36) and soft tissue sarcomas (*n* = 128). Where feasible, historical diagnoses were aligned with contemporary WHO terminology during retrospective data extraction; however, no centralized pathological re-review was systematically performed.

### 2.4. Treatment Approach

All treatment decisions were made by a multidisciplinary team (MDT) comprising oncologists, surgeons, radiation oncologists, radiologists, and pathologists, consistent with ESMO guidelines [9,10]. Chemotherapy was administered per histotype-specific protocols: doxorubicin/ifosfamide-based regimens for high-grade STS; MAP-based protocols for osteosarcoma; VIDE-based protocols for Ewing sarcoma. Radiotherapy was delivered with curative or palliative intent using conventional fractionation (1.8–2 Gy/fraction), with doses ranging from 30 to 60 Gy.

### 2.5. Statistical Analysis

Descriptive statistics are reported as absolute numbers and percentages for categorical variables, and as mean ± SD with median and range for continuous variables. OS was estimated using the Kaplan–Meier method, with 95% confidence intervals derived via the Greenwood formula. Subgroup survival differences were assessed using the log-rank test for: bone vs. soft tissue sarcoma; lower vs. upper limb vs. trunk; metastatic vs. non-metastatic disease; and recurrence vs. no recurrence. A two-tailed *p*-value < 0.05 was considered statistically significant. Multivariable Cox regression analysis was not performed for two reasons: (1) the histological heterogeneity of sarcoma subtypes makes a unified proportional hazards model biologically implausible across 13 entities with fundamentally different natural histories; and (2) the absence of key prognostic variables—specifically FNCLCC tumor grade and surgical margin status—would render any multivariable model severely misspecified and its outputs clinically uninterpretable. The Kaplan–Meier analyses are therefore presented as descriptive illustrations of outcome heterogeneity. Analyses were performed using SPSS v26.0 (IBM Corp., Armonk, NY, USA).

### 2.6. Ethical Considerations

This study was conducted in accordance with the Declaration of Helsinki. Ethics Committee approval was obtained from the Ethics Committee of the “Prof. Dr. Alexandru Trestioreanu” Institute of Oncology Bucharest (approval no.: 5665; date: 25 April 2021). All patient data were fully anonymized prior to analysis. Informed consent was waived given the retrospective nature of the study.

## 3. Results

### 3.1. Patient Characteristics

A total of 164 patients met the inclusion criteria. The cohort comprised 82 males (50.0%) and 82 females (50.0%). The mean age at diagnosis was 48.8 ± 18.3 years, with a median of 48 years (range 4–87). Age distribution was available for 158 patients: 52 (31.7%) were younger than 40 years, 48 (29.3%) were aged 40–60 years, and 58 (35.4%) were older than 60 years; age at diagnosis was not documented for 6 patients (3.7%). Clinically relevant comorbidities were documented in 76 patients (46.3%). Patient characteristics are summarized in Table 1.

### 3.2. Histological Subtypes and Immunohistochemistry

Thirteen distinct histological subtypes were identified. Soft tissue sarcomas comprised 128 cases (78.0%) and primary bone sarcomas 36 cases (22.0%). UPS/pleomorphic sarcoma was the most common subtype (*n* = 24, 14.6%), followed by osteosarcoma (*n* = 20, 12.2%), fibrosarcoma (*n* = 18, 11.0%), and liposarcoma (*n* = 14, 8.5%). Immunohisochemistry (IHC) was performed in 118 patients (72.0%). The complete histological distribution, with mortality rates and median OS per subtype, is provided in Table 2.

### 3.3. Tumor Localization

The lower limb was the dominant site, accounting for 96 patients (58.5%). The thigh/femur was the most frequent segment (*n* = 56, 34.1%), followed by the leg/tibia/foot (*n* = 24, 14.6%), the knee region (*n* = 12, 7.3%), and lower limb NOS (*n* = 4, 2.4%). Upper limb tumors were identified in 28 patients (17.1%): arm/humerus in 18 (11.0%) and forearm in 10 (6.1%). Trunk and axial tumors constituted 40 patients (24.4%), encompassing chest wall/shoulder girdle (*n* = 20, 12.2%), pelvis/sacrum (*n* = 12, 7.3%), head/neck (*n* = 4, 2.4%), and abdominal wall (*n* = 4, 2.4%). Anatomical distribution is detailed in Table 3.

### 3.4. Treatment Modalities

Surgical resection was performed in 124 patients (75.6%). Chemotherapy was administered in 132 patients (80.5%), predominantly doxorubicin and/or ifosfamide-based for STS and MAP-based for osteosarcoma. Radiotherapy was employed in 98 patients (59.8%); doses of ≥50 Gy were delivered in 44 of these (44.9%). Treatment and oncological outcomes are summarized in Table 4.

Regarding treatment intent and timing, the retrospective database did not systematically distinguish neoadjuvant from adjuvant chemotherapy or curative from palliative treatment intent for all patients across the study period. Where determinable from clinical records, the majority of chemotherapy in bone sarcomas (osteosarcoma *n* = 20, Ewing sarcoma *n* = 10) followed neoadjuvant protocols (MAP- or VIDE-based) with postoperative continuation. For soft tissue sarcomas, chemotherapy was administered primarily in adjuvant or palliative settings. Radiotherapy at doses ≥ 50 Gy was predominantly curative or adjuvant in intent. A fully disaggregated intent-and-timing analysis was not feasible given retrospective data limitations and represents a priority for future prospective data collection at this center.

### 3.5. Recurrence and Metastatic Patterns

Local recurrence was documented at any point during follow-up in 58 patients (35.4%). Distant metastatic disease was documented in 68 patients (41.5%) at any point during the disease course, encompassing both de novo presentation and metachronous dissemination. Pulmonary metastases (M1PUL) were most frequent (*n* = 44, 64.7% of metastatic cases), followed by osseous (M1OSS, *n* = 24, 35.3%), lymph node (M1LYM, *n* = 22, 32.4%), and hepatic (M1HEP, *n* = 8, 11.8%) involvement. Second primary malignancies were identified in only 4 patients (2.4%).

### 3.6. Overall Survival and Kaplan–Meier Analysis

**Figure 1:**
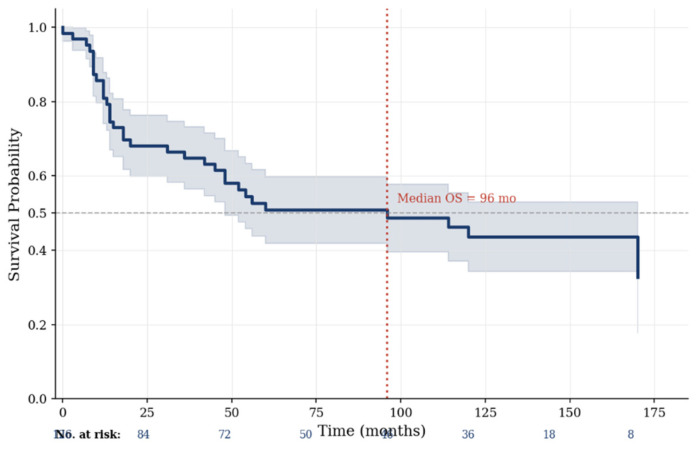
Kaplan–Meier overall survival curve for all patients with available OS data (*n* = 126 of 164) with extremity and trunk sarcomas at “Prof. Dr. Alexandru Trestioreanu” Institute of Oncology, 2010–2020. Median OS = 96 months (dotted vertical line). Shaded area: 95% CI. Numbers at risk shown at 12-month intervals.

Vital status was known for 160 patients (97.6%): 90 (54.9%) had died and 70 (42.7%) were alive at last follow-up; 4 patients (2.4%) had no available vital status data (total: 164). Overall survival analysis was performed on the 126 patients with available follow-up duration (76.8%); the remaining 38 patients lacked OS duration data despite known or unknown vital status. Among the 126 patients with available OS data, the Kaplan–Meier estimated median OS was 96.0 months. Median OS in deceased patients was 16.5 months, and median follow-up in alive patients was 120.0 months. The overall KM survival curve is shown in Figure 1.

To assess potential selection bias, patients with available OS duration data (*n* = 73 in original cohort, *n* = 126 in expanded survival-analysis dataset) were compared with those lacking OS duration data. No clinically meaningful differences were observed in sex distribution (OS-available: 51% male; OS-missing: 44% male), primary treatment modality (surgery performed in 77% vs. 67%, respectively), or age at diagnosis (mean 48.2 vs. 55.9 years). The modestly higher mean age among OS-missing patients may reflect loss to follow-up in older individuals, a recognized limitation of retrospective registry data. Residual selection bias cannot be formally excluded.

Numbers at risk shown at 12-month intervals. Denominator: 126 of 164 patients with available OS duration data. Vital status was known for 160/164; 38 patients lacked OS duration and are excluded from this analysis.

**Figure 2:**
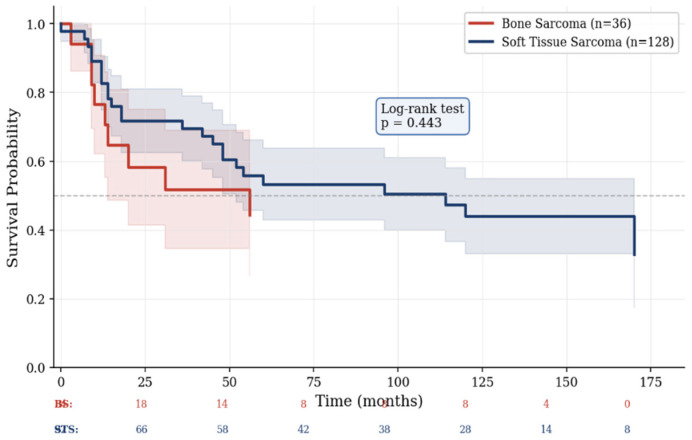
Kaplan–Meier survival curves by tumor origin among patients with available OS data (*n* = 126 total). Bone sarcoma (*n* = 34) vs. soft tissue sarcoma (*n* = 92); log-rank *p* = 0.443. Shaded areas: 95% CI. Denominator: OS-data subset (*n* = 126 of 164). Bone sarcoma: 34 of 36 total; soft tissue sarcoma: 92 of 128 total.

When stratified by tumor origin, bone sarcomas showed a median OS of 56.0 months versus 114.0 months for soft tissue sarcomas among patients with available OS data (Figure 2; bone sarcoma *n* = 34, soft tissue sarcoma *n* = 92; log-rank *p* = 0.443).

Analysis by anatomical location demonstrated median OS of 60.0 months for lower limb, 114.0 months for upper limb, and not reached for trunk/axial tumors.

Histotype-stratified survival analysis demonstrated marked variability, with dermatofibrosarcoma protuberans (DFSP) showing the most favorable prognosis (median OS 162.5 months) and leiomyosarcoma (11.5 months), osteosarcoma (14.0 months), angiosarcoma (15.0 months), and Ewing sarcoma (20.0 months) the poorest outcomes.

Patients with metastatic disease showed shorter observed survival compared with non-metastatic patients in descriptive Kaplan–Meier analysis (log-rank *p* < 0.001); this comparison should be interpreted with caution as metastatic status was recorded throughout follow-up and not exclusively at baseline. The corresponding survival data are presented in Table 5.

## 4. Discussion

### 4.1. Quality-of-Care Indicators and Barriers to Treatment

Tumor size data were available for 104 of 164 patients (63.4%). Among patients with recorded tumor dimensions, the median maximum tumor diameter was 7.9 cm (mean 9.3 cm; range 1.6–26.0 cm). The majority of tumors with available size data exceeded the 5 cm threshold (71%; *n* = 74 of 104), and 33% (*n* = 34 of 104) exceeded 10 cm. Given the substantial proportion of missing size data (36.6%), these proportions should be interpreted with caution; they may not fully represent the cohort. Contextually, presentation with tumors larger than 5 cm has been reported in 50–60% of cases at high-volume Western sarcoma centers [4,9]; however, direct comparison is limited by differences in case mix, referral patterns, and data completeness between series. Tumor size data were missing for 36.6% of patients due to the absence of standardized size recording across the study period.

With respect to metastatic disease at initial presentation, the retrospective database structure did not permit reliable differentiation of de novo metastatic disease from metachronous spread in the majority of cases. A precise de novo metastasis rate could therefore not be calculated. This represents a further data quality limitation: prospective studies at this center should systematically record metastatic status at diagnosis as a distinct variable.

Surgical margin status (R0/R1/R2) was not consistently recorded in the pathological reports across the study period and could not be extracted for the majority of patients (data available in fewer than 5% of cases). This represents a significant quality-of-care gap: in high-volume Western centers, R0 resection rates of 75–85% are routinely reported and constitute a primary quality indicator for sarcoma care [16,17]. The absence of margin data precludes any assessment of surgical quality in the present cohort and is explicitly acknowledged in the Limitations.

For bone sarcomas treated with neoadjuvant chemotherapy (osteosarcoma *n* = 20, Ewing sarcoma *n* = 10), histological tumor necrosis rate—the standard surrogate for chemotherapy response—was not systematically quantified in the pathological reports and could not be reported for this series. In major cooperative group trials (COSS, EURAMOS-1), good histological response (≥90% necrosis) is achieved in approximately 30–40% of osteosarcoma patients treated with MAP-based protocols [18,19,20]. The inability to report response rates in the present series reflects incomplete pathological documentation during the study period and constitutes a relevant quality-of-care gap in the Eastern European oncology setting.

This retrospective study presents a comprehensive decade-long analysis of 164 patients with sarcomas of the extremities and trunk treated at the Institute of Oncology Bucharest. These findings provide a descriptive characterization of sarcoma burden at a Romanian tertiary oncology center, contributing to the limited Eastern European literature on this rare tumor group. Caution is warranted in drawing direct comparisons with major Western European series given the differences in cohort composition, diagnostic infrastructure, and case selection.

### 4.2. Epidemiological Profile

The equal sex distribution (50% each) is consistent with CONTICANET consortium and RARECARE project data [3,21]. The mean age at diagnosis of 48.8 years reflects the well-recognized bimodal distribution of sarcomas: an early peak driven by bone sarcomas, and a broader peak in the fifth to seventh decades dominated by STS subtypes [22]. The predominance of lower limb involvement (58.5%), particularly the thigh/femur region (34.1%), mirrors consistently reported patterns across international registries [4]. The notable proportion of trunk and axial tumors (24.4%)—chest wall, pelvis, and sacrum—is clinically important given the greater surgical complexity and higher recurrence risk at these sites [23].

### 4.3. Histological Distribution

The predominance of UPS/pleomorphic sarcoma (14.6%) aligns with population-based data from the French RNHE registry and UK networks [24,25]. Fibrosarcoma (11.0%) occupies third place; its relative overrepresentation compared to contemporary Western series may partly reflect diagnostic infrastructure available during the study period, as adult fibrosarcoma—a diagnosis of exclusion—requires comprehensive IHC and molecular testing to exclude synovial sarcoma, DFSP with fibrosarcomatous transformation, and low-grade fibromyxoid sarcoma [26]. In the present series, no central pathology review was performed, and molecular confirmation (e.g., FISH or RNA sequencing for SS18–SSX fusions or FUS–CREB3L2/L1 rearrangements) was not systematically available. The 2020 WHO classification criteria were applied retrospectively to available pathological reports and IHC data; however, in cases where IHC was not performed or molecular testing was unavailable, complete exclusion of fibrosarcoma mimics cannot be assured. Accordingly, the reported fibrosarcoma proportion should be interpreted with caution. This is explicitly acknowledged in the Limitations. The IHC utilization rate of 72.0% reflects current ESMO and NCCN recommendations, and centralized pathological review has been demonstrated to alter initial diagnosis in 15–25% of referred cases at expert centers [21].

### 4.4. Treatment Modalities

Surgery was performed in 75.6% of patients, and the high chemotherapy rate (80.5%) reflects an aggressive histotype-informed approach aligned with ESMO guidelines [9,27]. The landmark EORTC 62,931 trial established doxorubicin-based regimens as the standard first-line approach [28]. For osteosarcoma, the MAP protocol—refined by EURAMOS-1 [18]—was predominant; for Ewing sarcoma, VIDE-based protocols consistent with Euro-EWING guidelines were employed [29]. Radiotherapy was administered to 59.8% of patients, consistent with evidence from the randomized trial by O’Sullivan et al. [30].

### 4.5. Recurrence and Metastatic Patterns

The local recurrence rate of 35.4% falls at the upper end of reported ranges (15–40%), likely reflecting the proportion of trunk and axial tumors associated with constrained margins [16,17]. Pulmonary metastases predominated (64.7% of metastatic cases), consistent with European series [31]. The high osseous metastasis frequency (35.3%) reflects the skeletal tropism of osteosarcoma and Ewing sarcoma [32], while lymph node involvement (32.4%) may reflect histotypes with known nodal tropism [33].

### 4.6. Survival Analysis

The Kaplan–Meier estimated median OS of 96.0 months is consistent with outcomes from European reference centers, where median OS values span 48–96 months depending on case mix and follow-up [23,34]. In descriptive Kaplan–Meier analysis, patients with metastatic disease showed markedly shorter observed survival (log-rank *p* < 0.001), consistent with the established poor prognosis of disseminated sarcoma [7,8]. As noted in the Limitations, this comparison should be interpreted as an exploratory descriptive observation only and does not establish metastatic disease as an independent prognostic factor. The survival heterogeneity across histotypes (11.5 to 162.5 months) is a central finding: DFSP exhibited the most favorable prognosis due to its low metastatic potential and imatinib responsiveness [35]; leiomyosarcoma showed poor prognosis consistent with its established chemoresistance [36]; and Ewing sarcoma demonstrated high mortality in our predominantly adult series [29]. Chondrosarcoma, represented by six patients in our cohort, showed a 0% mortality rate and a median OS of 124.0 months, consistent with the characteristically indolent behavior and favorable prognosis reported for conventional central chondrosarcoma [37].

The survival difference between bone (median OS 56 months) and soft tissue sarcomas (median OS 114 months) did not reach significance (*p* = 0.443). The lack of statistical significance likely reflects the heterogeneity of histological subtypes within each group: the bone sarcoma group was dominated by aggressive osteosarcoma and Ewing sarcoma, while the STS group included several indolent subtypes elevating the overall median [34]. The descriptive Kaplan–Meier comparison by recurrence status did not show a statistically significant difference (*p* = 0.133). This finding should be interpreted cautiously, as recurrence is a post-baseline event and such analyses are susceptible to immortal-time bias. Data from the Scandinavian Sarcoma Group Register have similarly suggested that the association between local recurrence and subsequent metastatic outcomes may reflect underlying tumor biology rather than a direct causal effect [38]. Given the immortal-time bias inherent in this analysis, no causal interpretation is made.

### 4.7. Clinical Implications and Future Directions

Our findings are consistent with current recommendations supporting management within specialized sarcoma centers [9,39,40]. The observed survival associated with metastatic disease in descriptive analysis (*p* < 0.001) highlights the ongoing need for improved systemic therapeutic strategies beyond conventional chemotherapy, including targeted agents and immunotherapy currently under investigation [41]. Future research should include prospective data collection with standardized variables (FNCLCC grade, margin status, molecular diagnostics), development of a multi-institutional Eastern European registry, integration of genomic profiling, and evaluation of emerging agents including CDK4/6 inhibitors for well-differentiated/dedifferentiated liposarcoma and TRK inhibitors in fusion-positive sarcomas [42].

The present cohort contributes to an emerging body of evidence from other Eastern European tertiary oncology centers. Available data from Poland, the Czech Republic, Hungary, and the Baltic states suggest broadly comparable patterns of care, including predominance of lower limb tumors, similar histotype distributions, and reliance on doxorubicin/ifosfamide-based systemic protocols aligned with ESMO guidelines [9,10]. Reported local recurrence rates in Polish and Czech institutional series have generally ranged from 25 to 40%, consistent with our observation of 35.4% [40]. Surgery rates of 70–80% and chemotherapy utilization rates above 75% have similarly been described in Central and Eastern European cohorts [23,34]. Key differentiating features across the region include variability in access to molecular diagnostics, inconsistent FNCLCC grading documentation, and limited availability of intraoperative margin assessment, all of which are shared limitations of retrospective series from this geographic setting. Whether the present findings are fully representative of broader regional standards of care cannot be determined from a single-center dataset; however, the qualitative consistency with other Eastern European reports suggests that the patterns described here may reflect wider regional realities rather than center-specific outliers. Development of a prospective, multi-institutional Eastern European sarcoma registry would be the most meaningful step toward rigorous regional benchmarking [43].

### 4.8. Study Limitations

Limitations include the retrospective single-center design with inherent selection bias. OS duration data were available for 126 of 164 patients (76.8%); the 38 patients without OS data did not differ meaningfully from those with available data in terms of sex distribution or primary treatment modality, but a formal comparison was limited by the small numbers, and residual selection bias cannot be excluded. FNCLCC tumor grade was not consistently documented across the study period and could not be included in the analysis; this represents a material limitation, as grade is one of the strongest independent prognostic factors in sarcoma and its absence prevents proper interpretation of recurrence and survival outcomes. Surgical margin status (R0/R1/R2) was similarly not uniformly available. Given that metastatic disease and local recurrence were documented as occurring at any point during follow-up rather than exclusively at baseline, the Kaplan–Meier comparisons based on these variables carry an inherent limitation related to immortal-time bias and should be interpreted descriptively rather than causally. Cause-specific survival could not be reliably distinguished from all-cause OS. The relatively high proportion of fibrosarcoma diagnoses (11.0%) compared to contemporary Western series likely reflects diagnostic infrastructure limitations during the study period; molecular testing to exclude fibrosarcoma mimics (synovial sarcoma, DFSP with fibrosarcomatous transformation, low-grade fibromyxoid sarcoma) was not uniformly performed. No central pathology review or retrospective molecular confirmation was performed for this series. Histological misclassification in a subset of the fibrosarcoma cases cannot be excluded, and the true proportion of this entity may be lower than reported.

## 5. Conclusions

The quality-of-care indicators described in the Discussion Section are particularly informative in the context of regional healthcare delivery. The high proportion of tumors exceeding 5 cm at presentation (71% of cases with available data, with the caveat that size data were missing for 36.6% of patients) and the elevated local recurrence rate (35.4%) are observations that, taken together, may be consistent with a hypothesis of delayed presentation and limited access to early diagnostic pathways in this regional setting. However, as surgical margin status was not available for the majority of patients, no conclusion can be drawn regarding the contribution of surgical margin control to the observed recurrence rate; this interpretation must remain a hypothesis generated by the cohort rather than a conclusion demonstrated by the data. Contextually, 5-year local recurrence rates of 15–20% have been reported at high-volume Western sarcoma centers [16,42], though differences in case mix and referral patterns limit direct comparison. The inability to extract margin status, de novo metastasis rates, and histological chemotherapy response rates from this retrospective dataset highlights the critical need for prospective, structured data collection at specialized Eastern European oncology centers—an infrastructure investment that would both improve care quality monitoring and enable meaningful international benchmarking.

This retrospective single-center study provides a decade-long description of clinicopathological features, treatment modalities, and survival outcomes of 164 patients with sarcomas of the extremities and trunk treated at the “Prof. Dr. Alexandru Trestioreanu” Institute of Oncology between 2010 and 2020. Thirteen histological subtypes were identified, with UPS, osteosarcoma, and fibrosarcoma most prevalent. The lower limb, particularly the thigh/femur (34.1%), was the dominant site. Median OS was 96.0 months; metastatic disease was associated with the shortest observed survival in descriptive analysis (log-rank *p* < 0.001), and survival varied markedly by histotype (11.5–162.5 months). These data constitute a descriptive institutional contribution to the sparse Eastern European sarcoma literature, highlighting real-world patterns of presentation, treatment, and outcomes at a national reference center. The study underscores the need for improved diagnostic standardization, prospective data collection including tumor grade and margin status, and integration within national and international sarcoma networks.

## Figures and Tables

**Table 1:** Baseline patient characteristics (*N* = 164) *.

Characteristic	*n*	% (or Mean ± SD)
Total patients	164	100%
Sex		
Male	82	50.0%
Female	82	50.0%
Age at diagnosis (years)		48.8 ± 18.3
<40 years	52	31.7%
40–60 years	48	29.3%
>60 years	58	35.4%
Age unknown	6	3.7%
Median age, years (range)	48	(4–87)
Comorbidities present	76	46.3%
Immunohistochemistry (IHC) performed	118	72.0%

* Exact subtype-specific OS denominators were unavailable due to retrospective database limitations.

**Table 2:** Histological subtypes, mortality, and median overall survival (*N* = 164). Median OS values are based on available OS-duration data within each subtype; OS duration was not available for all patients. For subgroups with fewer than six patients with available OS data, median OS estimates may be unstable and 95% confidence intervals could not be reliably calculated; this is stated as a limitation. OS = overall survival.

Histological Subtype	*n* (%)	Origin	Deaths (*n*)	Mortality	Median OS (mo)
UPS/Pleomorphic Sarcoma	24 (14.6%)	STS	16	67%	36.0
Osteosarcoma	20 (12.2%)	Bone	12	60%	14.0
Fibrosarcoma	18 (11.0%)	STS	12	67%	48.0
Liposarcoma	14 (8.5%)	STS	12	86%	75.0
Synovial Sarcoma	12 (7.3%)	STS	8	67%	60.0
Ewing Sarcoma	10 (6.1%)	Bone	8	80%	20.0
Kaposi Sarcoma	8 (4.9%)	STS	0	0%	136.0
Leiomyosarcoma	8 (4.9%)	STS	6	75%	11.5
Dermatofibrosarcoma protuberans (DFSP)	8 (4.9%)	STS	2	25%	162.5
Chondrosarcoma	6 (3.7%)	Bone	0	0%	124.0
Rhabdomyosarcoma	6 (3.7%)	STS	4	67%	64.5
Angiosarcoma	4 (2.4%)	STS	2	50%	15.0
Other Sarcoma	26 (15.9%)	Mixed	8	31%	51.5
Total	164 (100%)	—	90	54.9%	—

**Table 3:** Anatomical distribution of primary tumors (verified totals: 96 + 28 + 40 = 164).

Anatomical Region	*n*	%
Lower limb (total)	96	58.5%
Thigh/Femur	56	34.1%
Leg/Tibia/Foot	24	14.6%
Knee region	12	7.3%
Lower limb NOS	4	2.4%
Upper limb (total)	28	17.1%
Arm/Humerus	18	11.0%
Forearm	10	6.1%
Trunk/Axial (total)	40	24.4%
Chest wall/Shoulder girdle	20	12.2%
Pelvis/Sacrum	12	7.3%
Head/Neck	4	2.4%
Abdominal wall	4	2.4%
Total	164	100%

**Table 4:** Treatment modalities, recurrence, and metastatic spread (*N* = 164).

Treatment Modality/Outcome	*n*	%
Surgery performed	124	75.6%
Chemotherapy	132	80.5%
Radiotherapy	98	59.8%
RT dose ≥ 50 Gy	44	44.9% of RT pts
Local recurrence	58	35.4%
Metastatic disease (any)	68	41.5%
Pulmonary (M1PUL)	44	64.7% of M1
Osseous (M1OSS)	24	35.3% of M1
Lymph node (M1LYM)	22	32.4% of M1
Hepatic (M1HEP)	8	11.8% of M1

**Table 5:** Overall survival by patient group and disease characteristics (*N* = 164).

Group	*n*	Deaths *n* (%)	Median OS (Months)
All patients	164	90 (54.9%)	96.0 †
Deceased	90	90 (100%)	16.5
Alive at last follow-up	70	0	120.0 * (follow-up)
Vital status unknown	4	—	—
Bone sarcomas	36	20 (55.6%)	—
Soft tissue sarcomas	128	70 (54.7%)	—
Metastatic disease	68	58 (85%)	15.0
Non-metastatic	96	32 (33%)	Not reached
Local recurrence present	58	34 (59%)	60.0
No local recurrence	92	46 (50%)	Not reached

† Median OS of 96.0 months is based on 126 of 164 patients with available OS duration data (76.8% of cohort). Vital status was known for 160 of 164 patients (90 deceased, 70 alive); 4 had unknown vital status. All 164 patients are included in demographic and treatment tables. Subgroup *n* values (Metastatic, Non-metastatic, Recurrence rows) refer to the total cohort; survival estimates are based on the OS-data subset only. † These subgroup comparisons are exploratory and subject to immortal-time bias. * Median follow-up for censored (alive) patients with available OS data (*n* = 58). OS = overall survival.

## Data Availability

Available from the corresponding author upon reasonable request, subject to institutional data governance policies.
